# Finger Position Alters the Median Nerve Properties within the Carpal Tunnel: A Pre-Post MRI Comparison Study

**DOI:** 10.1371/journal.pone.0079273

**Published:** 2013-11-12

**Authors:** Mohammed Shaban Nadar, Mohsen H. Dashti, Jigimon Cherian

**Affiliations:** 1 Occupational Therapy Department, Faculty of Allied Health Sciences, Kuwait University, Jabriah, Kuwait; 2 Radiologic Sciences Department, Faculty of Allied Health Sciences, Kuwait University, Jabriah, Kuwait; 3 Department of Clinical Radiology, Al-Sabah Hospital, Ministry of Health, Kuwait; Northwestern University Feinberg School of Medicine, United States of America

## Abstract

**Purpose:**

The purpose of this study was to compare the properties of the median nerve and the flexor retinaculum within the carpal tunnel with Magnetic Resonance Imaging (MRI) under two conditions: (a) fingers extended, and (b) fingers in an isometric squeeze grip.

**Methods:**

Thirty-Four volunteers participated in this experimental study. The flexor retinaculum and median nerve characteristics were measured during both conditions using MRI.

**Results:**

The isometric squeeze grip condition resulted in significant palmar bowing of the flexor retinaculum (t = 7.67, *p*<.001), a significant flattening-ratio of the median nerve (t = 4.308, *p*<.001), and no significant decrease in the cross-sectional area of the median nerve (t = 2.508, *p* = 0.017).

**Conclusion:**

The isometric squeeze grip condition resulted in anatomical deformations within the carpal tunnel, possibly explained by the lumbrical muscles incursion into the carpal tunnel during finger flexion.

## Introduction

Carpal Tunnel Syndrome (CTS) is common neuropathy disorder encountered by healthcare specialists and is caused by prolonged abnormal force exerted on the median nerve within the Carpal Tunnel (CT) [1], [2]. The CT is a narrow space that contains nine tendons, their sheaths, and the median nerve. The position of the wrist and fingers has been shown to increase the force exerted on the median nerve and can therefore lead to possible impingement of the nerve. When compared to neutral position of the wrist, for example, the pressure in the CT during wrist flexion increases significantly [2]–[5] and is reported in one study to increase up to 2.5 times [6].

The most common conservative treatment of CTS involves splinting the wrist in neutral position while leaving the digits free. The rationale for this splint is that it maintains the CT in its widest position which allows the median nerve to heal. This intervention is used extensively by hand therapists and is considered adequate to improve the symptoms of CTS, although the evidence supporting significant benefits from traditional splints is limited [1], [7]–[9] and surgical intervention is reported to be as a superior treatment option for CTS than splinting (see O'Connor et al [8] & Verdugo et al [9] for reviews).

Several empirical studies implicated squeeze grip as one plausible factor that can limit the effectiveness of traditional splints in treating CTS, and cadaver studies confirmed that finger flexion leads to significant increases in CT pressure [10]–[13]. Another study reported that the pressure in the CT during active grip and neutral wrist to be higher than when the wrist was in extension and fingers relaxed [3]. Clinical findings have further correlated isometric squeeze grip with producing positive CTS symptoms is some patients [14], [12] Indeed, this finding became known as the “Closed Fist Test” of CTS [14]. This cumulative evidence indicates that finger movement directly increased the force exerted on the median nerve within the CT, which may contribute to median nerve compression. Based on such evidence and clinical observations, our objectives for this study were to measure the effects of finger position (isometric squeeze grip vs. finger extension) on the properties of median nerve within the CT. The properties measured in this project, using standardized high resolution MRI imaging, were (a) the flattening ratio, (b) median Nerve Area (MNA), (c) the pressure angle of the median nerve, and (d) the palmar bowing of the flexor retinaculum.

The flattening ratio is the most commonly applied measure of median nerve shape, and is reported to be stressed with CT pressure [15]. Flattening ratios involve quantifying the shape of the median nerve in approximation to an ellipse by measuring the ratio of the major axis to the minor axis [15]–[17], that is, the ratio of the medio-lateral diameter to the antero-posterior diameter on cross-sections. A ratio of 1 would indicate a round structure. The MNA is a measure of the cross-sectional area of the nerve [6], [15]–[20], and has been shown to decrease secondary to increased pressure within the CT [16], [19]. The pressure angle estimates the orientation of the median nerve relative to the flexor tendon and CT, and is also reported to shift in response to pressure variations within the CT [18], [21]. The palmar bowing of the flexor retinaculum is a measure used to quantify the internal pressure exerted on the retinaculum from the content within the CT [15], [18], [20]. Exaggerated palmar bowing of the flexor retinaculum has been reported to predict a diagnosis of CTS [6], [20].

The objective of this study was to compare the effects of finger position (finger extension versus isometric squeeze grip) on anatomical alterations within the CT. Our hypothesis was that, when compared to full finger extension, isometric squeeze grip would result in significant alterations within the CT space-content which would affect the properties of the most delicate structure within the CT, namely the median nerve [6]. More specifically, we anticipated that in comparison to finger extension, we would observe a significant decrease in the flattening ratio and MNA, an increased shift in the pressure angle of the nerve, and an increase in flexor retinaculum bowing toward the palmar aspect of the wrist.

The choice of MRI as the imaging tool in this study was based on the fact that MRI is a non-invasive and safe imaging method that produces high-quality and reliable images. Delineation of the median nerve is easy and we can accurately measure the cross-sectional areas of the nerve. The use of MRI in imaging and measuring the properties of the CT is relatively common and has been consistently performed in numerous studies. MRI can be reliably performed to image the CT in various wrist and the fingers positions, including full fingers extension and isometric squeeze grip [6], [15], [16], [18], [21], [22]. MRI has also been shown to be of value in the diagnosis of CTS [18]. Ultrasonography can produce images of sufficient reliability to measure the median nerve properties. However, the resolution of ultrasonography is less than that of MRI and is not suitable to measure higher level data pertinent to the median nerve, such as the flexor retinaculum bowing and the pressure angle of the median nerve.

Nerve conduction velocity measures were not considered because they do not produce informative data on the shape and pressure angle of the median nerve. In addition, Nerve conduction velocity measures produce data related to the quality of electrical transmission of the nerve, which usually result from either severe traumatic injury or prolonged compression of the median nerve, both of which do not match the immediate (live) effects of isometric squeeze grip and finger extension.

## Materials and Methods

### Ethics Statement

This study was reviewed and approved by Kuwait University Research and Human Ethics Committee and the procedures we followed were in accord with the standards of the committee. Signed informed consent was obtained from each participant after receiving an explanation of the study.

### Subjects

Forty healthy volunteers were recruited from the local community to participate in the study. Six participants were dropped from the study because their MRI images were glared and not clear for analysis, mainly due to hand movements during imaging. Consequently, a total of 34 participants were included in the study. Because the purpose of this project was to compare anatomical alterations between finger extension and isometric squeeze grip in the normal CT area, participants with known factors resulting in increased content of the CT such as Arthritis, fluid retention in pregnancy, Hyperthyroidism, Carpal Tunnel Syndrome, traumatic changes, and prior injury to the wrist or CT problems were excluded from the study.

We conducted a power analysis using G*Power version 3.0.1 software (downloaded from http://www.psycho.uni-duesseldorf.de/abteilungen/aap/gpower3/). The post-hoc power analysis for paired sample t-test and the analysis yielded a power of 84% for flattening ratio (group 1: mean = 2.27, SD = 0.34; group 2: mean = 2.64, SD = 0.76, r = 0.6, α = 0.01) and 99% for palmar bowing (group 1: mean = 2.27, SD = 0.93; group 2: mean = 3.34, SD = 1.13, r = 0.7, α = 0.01) demonstrating that we have enough power to reach our conclusion.

### Procedure

The participants were positioned prone and head-first with the participant's hand extended overhead. The measurements were performed with the elbow extended, forearm pronated, and the wrist in neutral position (0 degree angle). The wrist was supported by a static splint to immobilize the wrist throughout the imaging process. Based on the two conditions begin compared, participants were instructed to maintain their fingers in two different positions for MRI imaging; (a) fingers in full extension and (b) fingers in a forceful isometric squeeze grip. A five minute rest period between the two imaging positions was allowed. The order of imaging conditions was counterbalanced across participants where 20 participants started with the isometric squeeze grip followed by finger extension, and the other half started with finger extension followed by the isometric squeeze grip. The hands were imaged by using a General Electric 3T MRI scanner. The imaging started at the proximal radio-ulnar joint and was extend distally throughout the CT until the carpometacarpal joints level, with a 3 mm cross-sectional thickness, fat suppression sequence. The MRI technique that was used is standardized and reproducible as specified in the literature [6], [15], [18].

### Outcome measures

We used MRI to objectively assess a variety of measures pertaining to the median nerve properties at the hook of hamate level (i.e., flattening ratio, MNA, pressure angle of the median nerve and the flexor retinaculum bowing). A 2D axial section of the CT was selected at the level of hook of hamate from each MRI scan.

The flattening ratio was calculated as specified in the literature by dividing the major axis of the nerve by its minor axis [15]–[17]. To determine the median nerve cross-section area (MNA), the boundaries of the median nerve were manually traced by using the tools available within the MRI system software. The MNA was calculated in square millimeters, assuming an elliptical shape. The pressure angle of the median nerve within the CT was measured as described by Somay et al [18]. The pressure angle was formed by connecting the end of the perpendicular line (which measures the flexor retinaculum bowing) and the beginning of the trapezium tubercle - hook of hamate strait line. To measure the palmar bowing (also called displacement) of the flexor retinaculum, we drew a straight linear line to unite the hook of hamate with the trapezium and then measured (in millimeters) the perpendicular distance from the linear line between the hook of hamate and trapezium to the most palmar point (apex) of the transverse carpal ligament [15], [23].

The images were analyzed by two independent healthcare professionals who were blinded to the finger position condition by using the software impeded within the MRI machine. We used Lin's Concordance Correlation Coefficient (SPSS Macro) to calculate the agreement between the two raters in assessing the target variables [24]. We analyzed the data for normality by using quantile-quantile plots and Levene's test of equal variances. The data was found to satisfy parametric measurement conditions. The primary hypothesis was analyzed by using paired t-tests with a significant p-value<.05. A Bonferroni correction for 4 combinations of factors was adapted. Thus, p-values <.013 (0.05/4) were considered significant for each variable. All analyses were conducted using SPSS (Statistical Package for the Social Sciences) software version 19.

## Results

Thirty four subjects participated in this study, of whom 22 were males and 12 were females, with a mean age of 23.7 (SD: 6.6) years (range: 16–37). See table 1 for participant characteristics. The concordance correlation coefficients for the target variables assessed by the two raters ranged from 0.926 to 0.955, representing a moderate to substantial agreement between the two raters [25].

**Table 1:** Descriptive statistics of participants (n = 34).

	Minimum	Maximum	Mean	SD
Age (yrs.)	16	37	23.71	6.58
Height (cm)	153	179	170.35	7.63
Weight (Kg)	43	117	78.71	18.91
BMI	18.40	39.60	26.85	5.36
Education (yrs.)	10	20	14.0	2.87

Note: BMI  =  Body Mass Index.

The flattening ratio of the median nerve at the level of hook of hamate was 2.27 (SD = .34) for finger extension and 2.64 (SD = .76) for the isometric squeeze grip. The paired sample *t*(33) = 4.308, *p*<.001, indicated a significant reduction of the median nerve flattening ratio during the squeeze grip condition compared to finger extension. The mean MNA at the hook of hamate level was 8.74 mm^2^ (SD = 1.48) for finger extension and 8.41 mm^2^ (SD = 1.12) for the squeeze grip. The paired sample *t*(33) = 2.508, *p* = 0.017, showing a non-significant decrease in MNA with finger extension compared to isometric squeeze grip. The change in pressure angle of the median nerve was nearly negligible between the two conditions (*p*>.05). The mean palmar bowing of the flexor retinaculum during finger extension and isometric squeeze grip was 2.27 mm (SD = 0.93) and 3.34 mm (SD = 1.13), respectively. This shows that the palmar bowing of the flexor retinaculum was significantly greater during the isometric squeeze grip condition *t*(33) = 7.67, *p*<.001. All the *p*-values reported are after applying Bonferroni corrections to adjust for multiple comparisons. A summary of the results is presented in table 2.

**Table 2:** Summary of the anatomical changes within the Carpal canal.

	Finger extension mean (SD)	Isometric squeeze grip mean (SD)	Difference	Percent difference	*t*	df	Significance*
Flattening ratio	2.27 (0.34)	2.64 (0.76)	0.37	16.2%	4.308	33	*p*<.001
MNA	8.74 mm^2^ (1.48)	8.41 mm^2^ (1.12)	0.33 mm^2^	3.77%	2.508	33	*p* = .017
Pressure angle	26.3 (3.5)	25.9 (4.2)	0.4	1.5%	0.321	33	*P* = .75
Palmar bowing	2.27 mm (0.93)	3.34 mm (1.13)	1.07 mm	47.1%	7.67	33	*p*<.001

Note: MNA =  Median Nerve Area.

* Significant at *p*<.013 after a Bonferroni correction for 4 combinations of factors.

## Discussion

In this study, we compared the properties of the median nerve and transverse carpal ligament during finger extension and isometric squeeze grip. Our data showed that performing an isometric squeeze grip resulted in significant variations within the CT area that had direct effects on the median nerve, specifically a significantly flatter median nerve ratio and a significant palmar bowing of the flexor retinaculum.

The flattening ratio of the median nerve has been documented to vary with wrist position and to increase post CT release surgeries. Our results in this study demonstrated that isometric squeeze grip resulted in a similar fattening ratio pattern of the median nerve when stressed by, for example, wrist position (i.e., flexion [21], [16], [26]) or by a medical condition (i.e., Carpal Tunnel Syndrome [18]). That is, the mean flattening ratio of the median nerve in our subjects decreased during isometric squeeze grip relative to the finger extension position. Our findings are consistent with the findings of Kunze et al [16] who reasoned that the squeeze grip increase the nerve pinch around the median nerve boundary which indicate a local impingement on the median nerve from the adjacent tendons. This deformation of the median nerve during the isometric squeeze grip may potentially contribute to CTS. This may explain the higher incidence rate of CTS among individuals whose occupations require regular isometric squeeze griping, which have been reported to increase the risk of CTS more than double [27].

Although not significant, the decrease in size of the MNA during isometric squeeze grip relative to the fingers extended position may indicate impingement of the median nerve from surrounding structures. A similar conclusion was reported by Kunze et al (2010) [16] study which showed that the total percent of structures adjacent to the median nerve increased from fingers extension to isometric squeeze grip from 24% to 57%. The authors reasoned that the squeeze grip caused a local impingement on the median nerve from the adjacent tendons, which may eventually cause nerve deformation to be associated with CTS.

The marked increase in palmar displacement of the flexor retinaculum during isometric squeeze grip (relative to fingers extension) found in our study reflects an increased content or pressure within the CT area. Increased palmar bowing of the flexor retinaculum is cited as a constant finding in CTS patients [28], [29] and is regarded as predictive in diagnosing of CTS [6], [20].

Several studies documented changes in the pressure angle of the flexor tendons and median nerve during wrist movement. In one study, the tendons were generally similar in neutral and extended wrist positions, but were different with the wrist flexed [30]. Goetz et al (2010) [21] reported a significant difference in median nerve pressure angle with wrist flexion compared to neutral. Similar variations in median nerve orientation from wrist flexion compared to neutral were also reported by Kunze et al [26 & 16]. In another study of CTS patients, the pressure angle was significantly lower in CTS wrists than in control wrists, and the pressure angle was markedly improved eight weeks after surgery compared to pre-operative values [18]. In our study, however, we did not observe a significant change in pressure angle of the median nerve with different finger positions, demonstrating relatively unchanged nerve and tendon arrangement during finger movements when the wrist is maintained in neutral. Nonetheless, finger flexion was reported to cause additional tendon movement and nerve deformation when the wrist was flexed [21].

It has been suggested in several studies that median nerve properties can be used as indicators of disease severity [15], [18]. The changes found in our study as a result of isometric squeeze grip mildly replicated some of the morphological changes to the median nerve properties described with CTS patients. These changes during squeeze grip indicate increase content in the CT. The Lumbrical muscles can be the factor that explains the variations in median nerve and flexor retinaculum when forming a squeeze grip. The lumbricals are intrinsic muscles of the hand that originate from the long finger flexor tendon and function in flexing the metatarsophalangeal joints and extending the interphalangeal joints. The literature confirmed lumbrical muscle incursion into the narrow CT space during isometric squeeze grip [10]–[13] This can directly cause an increase in the content and volume of the CT and presumably increases pressure, consequently, narrowing the space available for the median nerve.

The cumulative evidence from our results compiled with the supporting literature implicate isometric squeeze grip with CTS. The effectiveness of traditional splints which restrict the wrist movement (but not finger movement) has been shown to provide partial success for relieving CTS symptoms to the median nerve [31], [32]. Since the currently prescribed splint for treating CTS immobilizes the wrist only and does not incorporate the fingers, we propose modifying the splint by extending its distal boarder to the crease of the metacarpophalangeal joints to restrict fingers flexion as well. Hypothetically, this modification should alleviate the symptoms of CTS more effectively than traditional splints. This, of course, remains to be empirically proven.

Our data demonstrate the presence of statistically significant changes in the properties of the median nerve during isometric squeeze grip. Our finding add further support to previous research which correlate squeeze grip with CTS [10]–[14], [19], [22], [27]. The evidence also suggest that fabricating a splint which prevents the fingers from flexing may be more successful in alleviating the symptoms of CTS than the currently prescribed splint which only restricts wrist movements. The proposed splint may in turn contribute to decompressing the median nerve. Future study directions should include controlled clinical trials to study the effectiveness of modified splints on treating patients with CTS. We are currently planning such clinical trial.

A limitation of this study is that the force of the isometric squeeze grip was not standardized across participants, which may be a potential confounder in the study. Another limitation is that measuring the properties of the median nerve relied on manually tracing the nerve by using the tools available within the MRI system software. The reliability of manual tracing may not be very high which could have affected the results of this study. Since our study was not designed to infer an etiological cause for CTS and was therefore unable to determine whether finger position is related to the development of CTS, a large scale clinical trial should be carried out to test the effectiveness of the proposed splint design that limit the wrist and fingers Metacarpophalangeal joints from flexing on relieving the symptoms of CTS.

## Conclusion

In this study, we objectively measured the effects of finger position on median nerve and flexor retinaculum properties. Performing an isometric squeeze grip resulted in statistically significant variations within the CT area that had direct effect on the median nerve, namely a significantly flatter median nerve ratio and a significant palmar bowing of the flexor retinaculum. The changes in the cross sectional area and the pressure angle of the median nerve were not significant
